# Sevelamer Use in End-Stage Kidney Disease (ESKD) Patients Associates with Poor Vitamin K Status and High Levels of Gut-Derived Uremic Toxins: A Drug–Bug Interaction?

**DOI:** 10.3390/toxins12060351

**Published:** 2020-05-27

**Authors:** Lu Dai, Björn K. Meijers, Bert Bammens, Henriette de Loor, Leon J. Schurgers, Abdul Rashid Qureshi, Peter Stenvinkel, Pieter Evenepoel

**Affiliations:** 1 Division of Renal Medicine and Baxter Novum, Department of Clinical Science, Intervention and Technology, Karolinska Institutet, 141 86 Huddinge, Stockholm, Sweden; lu.dai@ki.se (L.D.); tony.qureshi@ki.se (A.R.Q.); 2 Department of Microbiology Immunology and Transplantation, Nephrology and Renal Transplantation Research Group, KU Leuven-University of Leuven, B-3000 Leuven, Belgium; Bjorn.meijers@uzleuven.be (B.K.M.); bert.bammens@uzleuven.be (B.B.); jetty.deloor@uzleuven.be (H.d.L.); 3 Department of Nephrology, University Hospitals Leuven, B-3000 Leuven, Belgium; 4 Department of Biochemistry, Cardiovascular Research School Maastricht, Maastricht University, 6200MD Maastricht, The Netherlands; l.schurgers@maastrichtuniversity.nl

**Keywords:** uremic toxins, sevelamer, microbial metabolism, vitamin K, end-stage kidney disease

## Abstract

Gut microbial metabolism is not only an important source of uremic toxins but may also help to maintain the vitamin K stores of the host. We hypothesized that sevelamer therapy, a commonly used phosphate binder in patients with end-stage kidney disease (ESKD), associates with a disturbed gut microbial metabolism. Important representatives of gut-derived uremic toxins, including indoxyl sulfate (IndS), p-Cresyl sulfate (pCS), trimethylamine N-oxide (TMAO), phenylacetylglutamine (PAG) and non-phosphorylated, uncarboxylated matrix-Gla protein (dp-ucMGP; a marker of vitamin K status), were analyzed in blood samples from 423 patients (65% males, median age 54 years) with ESKD. Demographics and laboratory data were extracted from electronic files. Sevelamer users (*n* = 172, 41%) were characterized by higher phosphate, IndS, TMAO, PAG and dp-ucMGP levels compared to non-users. Sevelamer was significantly associated with increased IndS, PAG and dp-ucMGP levels, independent of age, sex, calcium-containing phosphate binder, cohort, phosphate, creatinine and dialysis vintage. High dp-ucMGP levels, reflecting vitamin K deficiency, were independently and positively associated with PAG and TMAO levels. Sevelamer therapy associates with an unfavorable gut microbial metabolism pattern. Although the observational design precludes causal inference, present findings implicate a disturbed microbial metabolism and vitamin K deficiency as potential trade-offs of sevelamer therapy.

## 1. Introduction

Hyperphosphatemia is one of the most common metabolic disorders in patients with chronic kidney disease (CKD) [1] and associates with adverse clinical outcomes across the stages of disease [2,3]. Phosphate binders, including calcium and non-calcium-containing agents, are a cornerstone in the treatment of hyperphosphatemia. In 2001, sevelamer hydrochloride was launched as the first non-metal-containing, nonabsorbable anion exchange binder. Currently, both sevelamer hydrochloride (HCl) and sevelamer carbonate are used in clinical practice. Sevelamer is a crosslinked polymer that exchanges HCl or carbonate for phosphate in the gastrointestinal tract. The HCl and carbonate moieties are absorbed from the gut, and the resulting phosphate-laden polymer is excreted in the feces [4]. Data from randomized clinical trials showed that, compared with calcium-containing phosphate binders (CCPB), sevelamer attenuated the progression of vascular calcification and conferred a survival benefit in prevalent and incident hemodialysis (HD) patients [5,6]. The non-specific binding of bile acids [7], uric acid [8], advanced glycation end products [9] and bacterial endotoxins [10] may contribute to the beneficial effects of sevelamer. Currently, sevelamer is the first-line phosphate binder in many dialysis units [11].

Sevelamer therapy, however, may also have some trade-offs. First, experimental and clinical evidence has suggested that sevelamer may bind useful and essential nutrients, such as vitamins D and K [12,13,14]. Second, gastrointestinal discomfort is common among sevelamer users, yet the underlying pathophysiology remains poorly defined. Third, depositions of mucosal sevelamer crystals [15,16,17] may alter the influx of nutrients and minerals in the colon and/or cause changes in their transit time. Altogether, they may trigger gut dysbiosis [18], ultimately resulting in an increased exposure to toxins, such as indoxyl sulfate (IndS), p-Cresyl sulfate (pCS), phenylacetylglutamine (PAG), and trimethylamine N-oxide (TMAO), which have been suggested to be precursors of various uremic complications and poor clinical outcomes in CKD [19,20,21,22,23]. Though this remains to be proven, it is unlikely that sevelamer affects hepatic phase-1 and -2 metabolisms.

Along with a recent increase in the awareness of drug–microbiome interactions [24], the present observational cohort study aimed to investigate whether sevelamer therapy associates with a poor vitamin K status and an adverse microbial metabolism profile in patients with ESKD.

## 2. Results

### 2.1. Baseline Characteristics

A total of 423 ESKD patients (median age 54 years old; 66% male; median serum phosphate, 4.6 mg/dL) were enrolled in the present analysis. Of these patients, 62% were treated with HD and 29% with peritoneal dialysis (median dialysis vintage, 24.6 months), and 37 patients (9%) were scheduled for preemptive renal transplantation. Sevelamer and CCPB were administered to 41% and 72% of the patients, respectively. Patients were classified according to sevelamer use, as shown in Table 1. Compared with sevelamer non-users, patients who were treated with sevelamer were characterized by younger age, higher BMI, lower albumin levels and higher phosphate, creatinine, IndS, TMAO, PAG and dp-ucMGP levels. No differences were found with regard to serum pCS levels, treatment modalities and other relevant drug therapy.

### 2.2. Univariate Correlations Between Sevelamer Use and Other Variables

In univariate Spearman rank correlation analysis, sevelamer use showed a positive correlation with BMI (rho = 0.14), cohort (rho = 0.26), creatinine (rho = 0.17), phosphate (rho = 0.21), IndS (rho = 0.22), TMAO (rho = 0.19), PAG (rho = 0.19) and dp-uc MGP (rho = 0.21), and a negative association with age (rho = −0.13) and albumin (rho = −0.10), as shown in Table S1.

### 2.3. Association Between Sevelamer Use and Uremic Toxins 

In multivariate linear regression, we investigated the associations between sevelamer use and serum uremic toxins levels. Sevelamer use was independently associated with a per one standard deviation (1-SD) increase in IndS (coefficient 0.28, *p* = 0.002, model R^2^ = 0.32), as shown in Table 2, and a per 1-SD increase in PAG (coefficient 0.20, *p* = 0.05, model R^2^ = 0.15), as shown in Table 3, after adjustments for age, sex, CCPB use, cohort, phosphate, creatinine and dialysis vintage. No associations were found between sevelamer use and pCS and TMAO levels, as shown in Table S2. In sensitive analyses, shown in Tables S3 and S4, sevelamer use was independently associated with a per 1-SD increase in IndS (coefficient 0.24, *p* = 0.05, model R^2^ = 0.23) but not with other uremic toxins in HD patients, (*n* = 261), and no significant association was observed in PD patients (*n* = 125).

### 2.4. Association Between Sevelamer Use and dp-uc MGP

In multivariate linear regression, we also investigated the association between sevelamer use and vitamin K status. After adjustments for age, sex, CCPB use, cohort, phosphate, creatinine and dialysis vintage, we found that sevelamer use was independently associated with a per 1-SD increase in dp-uc MGP (coefficient 0.36, *p* = 0.002, model R^2^ = 0.09), as shown in Table 4. In sensitive analyses, shown in Tables S3 and S4, sevelamer use was independently associated with a per 1-SD increase in dp-uc MGP both in HD patients (coefficient 0.29, *p* = 0.04, model R^2^ = 0.06; *n* = 261) and PD patients (coefficient 0.63, *p* = 0.005, model R^2^ = 0.13; *n* = 125).

### 2.5. AssociationsBetween dp-uc MGP and Uremic Toxins 

We further investigated the association between vitamin K status and serum uremic toxins levels. After adjustments for age, sex, sevelamer, CCPB, cohort, phosphate, creatinine and dialysis vintage, we found that an increased dp-uc MGP level was independently associated with a per 1-SD increase in PAG (coefficient 0.47, *p* < 0.0001, model R^2^ = 0.28) and TMAO (coefficient 0.16, *p* = 0.002, model R^2^ = 0.11), as shown in Table 5 and Table 6. No associations were found between dp-uc MGP and uremic toxins IndS and pCS levels, as shown in Table S5. In sensitive analyses, shown in Tables S3 and S4, an increased dp-uc MGP level was independently associated with a per 1-SD increase in PAG (coefficient 0.47, *p* < 0.0001, model R^2^ = 0.31) and TMAO (coefficient 0.19, *p* = 0.006, model R^2^ = 0.13) in HD patients (*n* = 261), and a significant association was observed among dp-uc MGP and PAG in PD patients (coefficient 0.29, *p* = 0.008, model R^2^ = 0.25; *n* = 125).

## 3. Discussion

The chief finding of this observational cohort study is that, in ESKD, sevelamer use independently associates with poor vitamin K status and increased serum levels of IndS and PAG. 

**Figure 1:**
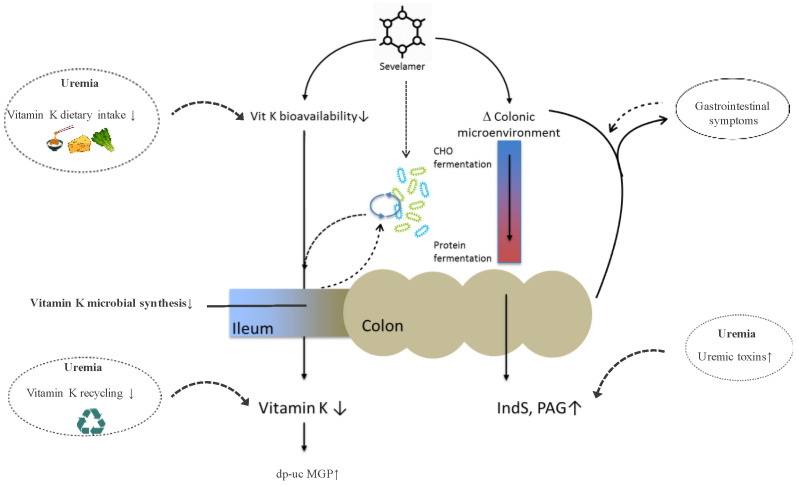
Sevelamer use and microbial metabolism in end-stage kidney disease. Abbreviations: dp-uc MGP, desphospho-uncarboxylated matrix Gla-protein; IndS, indoxyl sulfate; PAG, phenylacetylglutamine.

Vitamin K is the group name for a series of related compounds that share the ability to serve as cofactors for the microsomal enzyme, gamma-glutamyl carboxylase. The natural forms of vitamin K are vitamin K1 (phylloquinone) and vitamin K2 (menaquinone). Phylloquinone presents itself exclusively via the diet (leafy green vegetables and dairy produce), whereas menaquinones occur in food (dairy produce, natto and fermented products) as well as in the colon, where they are produced by the intestinal microflora. The extent to which the various nutritional and colonic menaquinones contribute to the biosynthesis of the various Gla-proteins is a matter of ongoing debate [20,21]. Experimental studies on the effects of the oral and colorectal administration of vitamin K on the concentration of circulating prothrombin in vitamin K deficient rats demonstrated that the bioavailability of colonic vitamin K is more than 50-fold lower than the bioavailability of oral vitamin K [25]. Conversely, data from germ-free rodents [26], and experimental and clinical studies with broad spectrum antibiotics [27,28,29], indicate that gut microbial metabolism is important to maintain adequate vitamin K stores in the mammalian host. The substantial production of menaquinones by microbiota inhabiting the terminal ileum may reconcile this apparent controversy, and this is further supported by a recent report suggesting that microbiome patterns have a significant impact on vitamin K status [30]. Vitamin K deficiency is highly prevalent in CKD [31]. Reduced vitamin K intake due to dietary restrictions [32] and impaired vitamin K recycling [33] may be the main causes of vitamin K deficiency in CKD. Mounting evidence indicates that vitamin K deficiency is involved in the pathogenesis of accelerated vascular calcification and bone fragility, both common features of the uremic phenotype [31,34,35]. In the present study, sevelamer users were characterized by a poor vitamin K status, which confirms and extends data from a recent smaller cohort study [13]. It is speculated that sevelamer sequesters vitamin K along the length of the gastrointestinal tract, and thereby hampers its absorption. However, studies assessing the binding of vitamin K (both phylloquinone and menaquinone) in vitro yielded inconsistent results [14,36]. Thus, uncertainty persists as to whether sevelamer interferes with vitamin K bioavailability or not. In the present study, we observed the significant and independent associations between poor vitamin K status and high serum levels of PAG and TMAO. Although this observation links gut dysbiosis and vitamin K deficiency, the observational design precludes firm conclusions both on the causality of the association and on the direction. Intriguingly, while gut dysbiosis may result in the decreased microbial synthesis of vitamin K, a low availability of vitamin K in the gut, alternatively, may disturb microbial composition and metabolism, as shown in Figure 1. Indeed, menaquinones are necessary growth factors to regulate human gut microbiota, such as in the *Faecalibacterium* species [37]. 

Uremia is characterized by the retention of uremic toxins, which can be categorized according to physicochemical characteristics affecting their behavior during dialysis, or according to their origin [38]. The gut is recognized as an important source of uremic retention molecules, with IndS, pCS, TMAO and PAG [22,23,39,40,41,42] being important representatives. Circulating levels of these toxins increase alongside the progression of CKD as a consequence of decreased elimination and increased generation, to reach levels in ESKD that are more than 10-fold higher than in healthy controls [43,44]. In the present study, sevelamer use was associated with high IndS and PAG levels, independent of classical and non-classical determinants including age, sex, phosphate, creatinine and dialysis vintage. This observation might come to a surprise since some, but not all, in vitro studies have shown the chelation of the precursor’s compounds by sevelamer [45,46]. However, sevelamer may disturb the colonic microenvironment, either by dragging nutrients and minerals into the colon and/or by prolonging the colon transit time, ultimately resulting in accentuated protein fermentation, as shown in Figure 1. While Brandenburg et al. [47] reported that eight weeks of sevelamer hydrochloride treatment did not change serum IndS and Indole-3-acetic acid (IAA), this treatment increased serum pCS levels in HD patients. In contrast, a randomized clinical trial in patients with CKD stages 3–4 [45] showed that 12-week sevelamer treatment did not change the serum levels of IS, pCS and IAA levels, compared with the placebo group. It should be emphasized that, in individuals with preserved renal function, serum toxin levels, as opposed to 24 h urine excretion rates, provide a much less sensitive readout of colonic microbial metabolism.

Our observation that sevelamer use is associated with an increased exposure to uremic toxins and poor vitamin K may represent the clinical-relevant trade-offs of sevelamer therapy and may explain the neutral, or even negative, findings in intervention trials [48,49]. Pharmaceutical agents have both beneficial and undesirable effects. Although many drugs have gastrointestinal side effects, and the gut microbiome itself is pivotal for human health, the role of the gut microbiota in these processes is rarely considered [24]. Importantly, drug-bug interactions may not only be limited to sevelamer. According to a recent survey, up to 25% of drugs may affect the microbiome, and a better knowledge of drug–bug interactions may open new paths for side effect control besides other benefits [24].

Several limitations of the present study are acknowledged. Firstly, given the observational cohort study design, we were not able to address the causality among sevelamer use and disturbed gut microbiota. Secondly, we acknowledge the possibility of residual confounding. Information on diet and residual renal function was missing. However, the levels of gut-derived toxins (pCS, TMAO and PAG) did not differ between patients with CCPB vs. patients free of CCPB, rendering residual confounding less likely. A major strength of the study was the availability of sevelamer medication data together with the profiles of uremic toxins IndS, pCS, TMAO and PAG, and vitamin K status in a fairly large and well-phenotyped cohort.

In conclusion, our data suggest that sevelamer use is associated with disturbed gut microbial metabolism, indicated by high serum IndS and PAG levels and poor vitamin K status in ESKD. Our observation conveys the message that nephrologists should not forget the drug–bug interactions, whereby sevelamer therapy could alter the colonic microbiome as a potential trade-off of improving phosphate control.

## 4. Materials and Methods 

### 4.1. Study Population

The study population comprised adult patients with ESKD referred for kidney transplantation in Leuven (*n* = 410, University Hospital Leuven, Belgium, between 23 April 2006 and 17 March 2016) and Stockholm (*n* = 117, Karolinska University Hospital Huddinge, Sweden, between 4 March 2009 and 17 June 2015). Patients treated either with non-calcium-containing phosphate binders other than sevelamer or vitamin K antagonists were excluded. The study was conducted in adherence to the Declaration of Helsinki and approved by the ethical committees of University Hospitals Leuven (S52091, approved on 31/03/2010) and Karolinska University Hospital (2008/1748-31/2, approved on 27/11/2008), respectively. Informed consent was obtained from each patient.

### 4.2. Clinical Data

Demographics, comorbidities, phosphate binders and other relevant medications of routine biochemistry were extracted from electronic files. 

### 4.3. Biochemical Measurements

Blood samples taken at the admission for the renal transplant procedure were random (non-fasting for unpredictable deceased donor transplant and fasting for living donor transplant). Samples were stored for <2 h at 5 °C until centrifugation. Upon arrival at the laboratory, the blood samples were centrifuged at 1900× *g* for 10 min, aliquoted, and either processed immediately (standard technique) or stored at −80 °C until analysis. Hemoglobin, creatinine, serum calcium, phosphate and albumin were measured using standard laboratory techniques. Intact parathyroid hormone (PTH) was determined by an automated second generation electro-chemiluminescence immunoassay on a Roche Cobas platform (Roche Diagnostics, GmbH, Germany). Serum levels of IndS, pCS, TMAO and PAG were quantified using a dedicated ultra-performance liquid chromatography–tandem mass spectrometry (UPLC-MS/MS) method as previously described [50].

Vitamin K status was assessed by measuring desphospho-uncarboxylated matrix Gla-protein (dp-uc MGP) in EDTA plasma using a commercial dual-antibody enzyme-linked immunoassay (InaKtif MGP, iSYS; IDS, Boldon, UK), as described [51]. The within-run and total variations of this assay were 0.8–6.2% and 3.0–8.2%, respectively.

### 4.4. Statistical Analysis 

Data are expressed as median (interquartile range, IQR), mean (standard deviation, SD), and number or percentage, as appropriate. Statistical significance was set at the level of *p* < 0.05. Comparisons between two groups were assessed with the non-parametric Wilcoxon test for skewed continuous variables, and the *t* test for normally distributed variables and Fischer´s exact test for nominal variables. Spearman rank correlation analysis was used to determine the associations between two variables. Multivariate linear regression analyses were performed to examine the associations between sevelamer use, uremic toxins levels and vitamin K status, as well as the association between vitamin K status and uremic toxins levels. Statistical analyses were performed using statistical software SAS, version 9.4 (SAS Campus Drive, Cary, NC, USA) and Stata 15.1 (Stata Corporation, College Station, TX, USA).

## Figures and Tables

**Table 1:** Baseline characteristics of patients according to sevelamer users and non-users.

	All Patients (*n* = 423)	Sevelamer Non-Users (*n* = 251, 59%)	Sevelamer Users (*n* = 172, 41%)	*p*-Value
Demography and clinical characteristics				
Age, years	54 (43–63)	56 (43–65)	52 (43–61)	0.01
Male sex, *n* (%)	277 (66%)	170 (68%)	107 (62%)	0.24
BMI, kg/m^2^	24.2 (22.0–26.6)	23.6 (21.5–26.0)	24.9 (22.5–27.8)	0.01
Diastolic BP, mmHg	80 (73–89)	80 (74–89)	79 (72–89)	0.38
Systolic BP, mmHg	140 (127–153)	140 (125–153)	140 (129–152)	0.96
Dialysis vintage, months	24.6 (6.1–42.6)	24.2 (9.4–41.1)	25.6 (1.7–49.8)	0.68
Treatment				0.82
Non-dialysis, *n* (%)	37 (9%)	22 (9%)	15 (9%)	
Hemodialysis, *n* (%)	261 (62%)	152 (60%)	109 (63%)	
Peritoneal dialysis, *n* (%)	125 (29%)	77 (31%)	48 (28%)	
Biochemical measurements				
Hemoglobin, g/dL	11.8 (1.6)	11.6 (1.6)	12.0 (1.6)	0.06
Creatinine, mg/dL	7.6 (5.9–9.3)	7.3 (5.4–8.8)	7.9 (6.4–10.2)	<0.001
Calcium, mg/dL	9.1 (8.5–9.6)	9.1 (8.5–9.6)	9.1 (8.6–9.7)	0.34
Phosphate, mg/dL	4.6 (3.8–5.6)	4.3 (3.6–5.3)	5.0 (4.1–5.9)	<0.001
Serum albumin, g/L	40.7 (36.0–45.1)	41.7 (37.0–45.4)	40.0 (35.0–44.8)	0.05
Parathyroid hormone, ng/L	168 (87–289)	166 (81–269)	173 (96–320)	0.11
Uremic toxins				
Indoxyl sulfate, μM	101 (62–151)	89 (55–136)	123 (76–161)	<0.001
p-Cresyl sulfate, μM	166 (112–230)	164 (108–230)	170 (119–226)	0.60
TMAO, μM	58 (34–99)	50 (29–91)	67 (44–120)	<0.001
Phenylacetylglutamine, μM	64 (34–103)	58 (28–88)	76 (44–120)	<0.001
Vitamin K status				
dp-ucMGP, pmol/L	1050 (712–1565)	952 (655–1353)	1180 (837–1832)	<0.001
Medications				
Ca-blocker, *n* (%)	144 (34.0%)	82 (32.7%)	62 (36.0%)	0.47
Beta-blocker, *n* (%)	205 (48.6%)	118 (47.2%)	87 (50.6%)	0.49
ACEi/ARB, *n* (%)	204 (48.3%)	124 (49.4%)	80 (46.8%)	0.60
Statin, *n* (%)	202 (47.8%)	125 (49.8%)	77 (44.8%)	0.31
PPI use, *n* (%)	144 (34.1%)	82 (32.7%)	62 (36.3%)	0.45
25-OH vitamin D use, *n* (%)	198 (46.8%)	127 (51.0%)	71 (41.0%)	0.06
CCPB, *n* (%)	303 (71.6%)	186 (74.1%)	117 (68.0%)	0.17

Data are presented as median (interquartile range, IQR) or mean (standard deviation, SD) for continuous measures, and *n* (%) for categorical measures. Abbreviations: BMI, body mass index; BP, blood pressure; TMAO, trimethylamine N-oxide; dp-ucMGP, desphospho-uncarboxylated matrix Gla-protein; ACEi/ARB, angiotensin-converting enzyme inhibitor/angiotensin II receptor blockers; PPI, proton pump inhibitors; 25-OH vitamin D, 25-hydroxy vitamin D; CCPB, calcium-containing phosphate binders.

**Table 2:** Multivariate linear regression analysis of the association between sevelamer use and serum IndS in 423 ESKD patients (R^2^ = 0.32).

	per 1-SD Increase in IndS	
	Coefficients	*p*-Value
Sevelamer use	0.28	0.002
per 1-SD increase in age	−0.03	0.43
Sex, male vs. female	−0.04	0.64
Cohort	−0.13	0.36
CCPB use	0.11	0.23
per 1-SD increase in phosphate	−0.03	0.53
per 1-SD increase in creatinine	0.49	<0.0001
per 1-SD increase in dialysis vintage	0.15	0.004

Abbreviations: IndS, indoxyl sulfate; ESKD, end-stage kidney disease; 1-SD, one standard deviation; CCPB, calcium-containing phosphate binder.

**Table 3:** Multivariate linear regression analysis of the association between sevelamer use and serum PAG in 423 ESKD patients (R^2^ = 0.15).

	per 1-SD Increase in PAG	
	Coefficients	*p*-Value
Sevelamer use	0.20	0.05
per 1-SD increase in age	0.11	0.02
Sex, male vs. female	−0.20	0.06
Cohort	−0.14	0.35
CCPB use	−0.05	0.66
per 1-SD increase in phosphate	−0.04	0.45
per 1-SD increase in creatinine	0.36	<0.0001
per 1-SD increase in dialysis vintage	0.11	0.05

Abbreviations: PAG, phenylacetylglutamine; ESKD, end-stage kidney disease; 1-SD, one standard deviation; CCPB, calcium-containing phosphate binder.

**Table 4:** Multivariate linear regression analysis of the association between sevelamer use and serum dp-uc MGP in 423 ESKD patients (R^2^ = 0.09).

	per 1-SD Increase in dp-uc MGP	
	Coefficients	*p-*Value
Sevelamer use	0.36	0.002
per 1-SD increase in age	0.23	<0.0001
Sex, male vs. female	−0.06	0.61
Cohort	0.13	0.46
CCPB use	−0.20	0.002
per 1-SD increase in phosphate	−0.007	0.90
per 1-SD increase in creatinine	0.12	0.05
per 1-SD increase in dialysis vintage	0.007	0.91

Abbreviations: dp-uc MGP, desphospho-uncarboxylated matrix Gla-protein; ESKD, end-stage kidney disease; 1-SD, one standard deviation; CCPB, calcium-containing phosphate binder.

**Table 5:** Multivariate linear regression analysis of the association between plasma dp-uc MGP and PAG in 423 ESKD patients (R^2^ = 0.28).

	per 1-SD Increase in dp-uc MGP	
	Coefficients	*p*-Value
per 1-SD increase in PAG	0.47	<0.0001
Sevelamer use	0.26	0.01
CCPB	−0.19	0.07
per 1-SD increase in age	0.18	<0.0001
Sex, male vs. female	0.04	0.71
Cohort	0.23	0.14
per 1-SD increase in phosphate	0.02	0.71
per 1-SD increase in creatinine	−0.05	0.39
per 1-SD increase in dialysis vintage	−0.03	0.57

Abbreviations: dp-uc MGP, desphospho-uncarboxylated matrix Gla-protein; PAG, phenylacetylglutamine; ESKD, end-stage kidney disease; 1-SD, one standard deviation; CCPB, calcium-containing phosphate binder.

**Table 6:** Multivariate linear regression analysis of the association between plasma dp-uc MGP and TMAO in 423 ESKD patients (R^2^ = 0.11).

	per 1-SD Increase in dp-uc MGP	
	Coefficients	*p*-Value
per 1-SD increase in TMAO	0.16	0.002
Sevelamer use	0.33	0.003
CCPB	−0.22	0.06
per 1-SD increase in age	0.21	<0.0001
Sex, male vs. female	−0.05	0.64
Cohort	0.11	0.54
per 1-SD increase in phosphate	−0.01	0.81
per 1-SD increase in creatinine	0.10	0.12
per 1-SD increase in dialysis vintage	−0.007	0.92

Abbreviations: dp-uc MGP, desphospho-uncarboxylated matrix Gla-protein; TMAO, trimethylamine N-oxide; ESKD, end-stage kidney disease; 1-SD, one standard deviation; CCPB, calcium-containing phosphate binder.

## Supplementary Materials

The following are available online at https://www.mdpi.com/2072-6651/12/6/351/s1, Table S1: Univariate correlations among sevelamer use and other variables, Table S2: Multivariate linear regression analysis of association among sevelamer use and pCS and TMAO in 423 ESKD patients, Table S3: Sensitive analysis of association between sevelamer use, uremic toxins and dp-ucMGP in HD patients (*n* = 261). Table S4: Sensitive analysis of association between sevelamer use, uremic toxins and dp-ucMGP in PD patients (*n* = 125). Table S5: Multivariate linear regression analysis of association among dp-ucMGP and IndS and pCS in 423 ESKD patients.
