# Supplementary material for: Sevelamer Use in End-Stage Kidney Disease (ESKD) Patients Associates with Poor Vitamin K Status and High Levels of Gut-Derived Uremic Toxins: A Drug–Bug Interaction?
Source: Toxins (Basel). 2020 May 27;12(6):351. doi: 10.3390/toxins12060351 (PMC7354623; doi:10.3390/toxins12060351)
Supplement: Supplementary file 1 [file toxins-12-00351-s001.pdf]

Supplementary Materials: Sevelamer Use in End-Stage Kidney Disease (ESKD) Patients Associates with Poor Vitamin K Status and High Levels of Gut-Derived Uremic Toxins: A Drug–Bug Interaction?

Lu Dai, Björn K. Meijers, Bert Bammens, Henriette de Loor, Leon J. Schurgers, Abdul Rashid Qureshi, Peter Stenvinkel and Pieter Evenepoel

Table S1. Univariate correlations among sevelamer use and other variables.

	Sevelamer Use	
**Variables**	**Rho**	***p*-Value**
Age	−0.13	0.01
Sex, male	−0.06	0.24
DBP, mmHg	−0.04	0.38
SBP, mmHg	0.002	0.96
BMI, kg/m^2	0.14	0.01
Cohort	0.26	<0.0001
Hemoglobin, g/dL	0.09	0.06
Creatinine, mg/dL	0.17	0.0003
Calcium, mg/dL	0.05	0.34
Phosphate, mg/dL	0.21	<0.0001
Albumin, g/L	−0.10	0.05
PTH, ng/L	0.08	0.11
Vintage, month	0.02	0.68
Indoxyl sulfate, µM	0.22	<0.0001
p-Cresyl sulfate, µM	0.03	0.60
TMAO, µM	0.19	<0.0001
PAG, µM	0.19	<0.0001
dp-ucMGP, pmol/L	0.21	<0.0001
CCPB use	−0.07	0.17

Abbreviations: DBP, diastolic blood pressure; SBP, systolic blood pressure; BMI, body mass index; PTH, parathyroid hormone; TMAO, trimethylamine N-oxide; PAG, phenylacetylglutamine; dp-ucMGP, desphospho-uncarboxylated matrix Gla-protein; CCPB, calcium-containing phosphate binder.

Table S2. Multivariate linear regression analysis of association among sevelamer use and pCS and TMAO in 423 ESKD patients.

	per 1-SD Increase of pCS*		per 1-SD Increase of TMAO#	
	**Coefficients**	***p*-Value**	**Coefficients**	***p*-Value**
Sevelamer use	0.13	0.23	0.15	0.16
per 1-SD increase of age	0.08	0.15	0.14	0.007
Sex, male vs female	0.18	0.11	−0.08	0.45
Cohort	−0.46	0.005	0.16	0.33
CCPB use	−0.07	0.52	0.06	0.61
per 1-SD increase of phosphate	−0.01	0.84	0.03	0.61
per 1-SD increase of creatinine	0.11	0.07	0.17	0.005
per 1-SD increase of dialysis vintage	−0.08	0.17	0.09	0.12

Abbreviations: pCS, p-Cresyl sulphate; TMAO, trimethylamine N-oxide; ESKD, end-stage kidney disease; 1-SD, one standard deviation; CCPB, calcium-containing phosphate binder.* R^2 = 0.05, # R^2 = 0.06.

Table S3. Sensitive analysis of association between sevelamer use, uremic toxins and dp-ucMGP in HD patients (n = 261).

	**1-SD IndS**	**1-SD TMAO**	**1-SD pCS**	**1-SD PAG**	**1-SD dp-ucMGP**	**1-SD dp-ucMGP**	**1-SD dp-ucMGP**	**1-SD dp-ucMGP**	**1-SD dp-ucMGP**
Sevelamer use	0.235*	0.109	0.0807	0.119	0.294*	0.229	0.272	0.287*	0.267
CCPB	0.177	0.102	−0.121	−0.0941	−0.233	−0.201	−0.276	−0.226	−0.254
per 1-SD increase of age	−0.0612	0.0967	0.0868	0.109	0.226**	0.185**	0.211**	0.217**	0.234**
Sex, male vs female	−0.0231	−0.113	0.227	−0.240	−0.0517	0.0620	−0.0490	−0.0738	−0.0498
Cohort	−0.0472	−0.0429	−0.664**	−0.0660	0.567*	0.630*	0.581*	0.624*	0.576*
per 1-SD increase of phosphate	−0.0593	0.0307	−0.0175	−0.0219	0.00632	0.0278	−0.000787	0.0108	0.0149
per 1-SD increase of creatinine	0.416***	0.201**	0.0608	0.353***	0.119	−0.0548	0.0811	0.110	0.0796
per 1-SD increase of dialysis vintage	0.0994	0.0581	−0.0995	0.0462	0.00660	−0.00765	−0.00560	0.0136	−0.00481
per 1-SD increase of PAG						0.474***			
per 1-SD increase of TMAO							0.186**		
per 1-SD increase of pCS								0.0949	
per 1-SD increase of IndS									0.0985
R^2	0.230	0.058	0.057	0.113	0.098	0.311	0.130	0.107	0.106

Abbreviations: dp-ucMGP, desphospho-uncarboxylated matrix Gla-protein; HD, hemodialysis; 1-SD, one standard deviation; IndS, indoxyl sulfate; TMAO, trimethylamine N-oxide; pCS, p-Cresyl sulfate; PAG, phenylacetylglutamine; CCPB, calcium-containing phosphate binder.* $p < 0.05$, ** $p < 0.01$, *** $p < 0.001$.

Table S4. Sensitive analysis of association between sevelamer use, uremic toxins and dp-ucMGP in PD patients (n = 125).

	**1-SD IndS**	**1-SD TMAO**	**1-SD pCS**	**1-SD PAG**	**1-SD dp-ucMGP**	**1-SD dp-ucMGP**	**1-SD dp-ucMGP**	**1-SD dp-ucMGP**	**1-SD dp-ucMGP**
Sevelamer use	0.242	0.0923	0.227	0.236	0.626**	0.553*	0.619**	0.655**	0.598**
CCPB	−0.0776	−0.138	−0.0383	−0.134	−0.367	−0.308	−0.357	−0.373	−0.357

per 1-SD increase of age	−0.0453	0.264**	0.0586	0.0895	0.299**	0.268**	0.283**	0.302**	0.305**
Sex, male vs female	−0.128	−0.187	0.128	−0.352*	−0.174	−0.0537	−0.157	−0.170	−0.157
Cohort	−0.0205	0.637*	−0.555	−0.0248	−0.285	−0.224	−0.328	−0.360	−0.287
per 1-SD increase of phosphate	0.0731	0.0356	0.0270	−0.129	−0.0155	0.0237	−0.0186	−0.00939	−0.0223
per 1-SD increase of creatinine	0.604***	0.185	0.212	0.620***	0.225	0.0390	0.213	0.247*	0.147
per 1-SD increase of dialysis vintage	0.118	0.0312	−0.0924	0.00855	−0.137	−0.113	−0.137	−0.154	−0.152
per 1-SD increase of PAG						0.290**			
per 1-SD increase of TMAO							0.0734		
per 1-SD increase of pCS								−0.104	
per 1-SD increase of IndS									0.127
R^2	0.485	0.131	0.079	0.314	0.190	0.245	0.195	0.200	0.198

Abbreviations: dp-ucMGP, desphospho-uncarboxylated matrix Gla-protein; PD, peritoneal dialysis; 1-SD, one standard deviation; IndS, indoxyl sulfate; TMAO, trimethylamine N-oxide; pCS, p-Cresyl sulfate; PAG, phenylacetylglutamine; CCPB, calcium-containing phosphate binder* $p < 0.05$, ** $p < 0.01$, *** $p < 0.001$.

Table S5. Multivariate linear regression analysis of association among dp-ucMGP and IndS and pCS in 423 ESKD patients.

	**per 1-SD Increase of dp-uc MGP***			**per 1-SD Increase of dp-uc MGP#**	
	**Coefficients**	***p*-Value**		**Coefficients**	***p*-Value**
per 1-SD increase of IndS	0.12	0.06	per 1-SD increase of pCS	0.06	0.26
Sevelamer use	0.32	0.005	Sevelamer use	0.35	0.002
CCPB	−0.21	0.06	CCPB	−0.19	0.09
per 1-SD increase of age	0.23	<0.0001	per 1-SD increase of age	0.22	<0.0001
Sex, male vs. female	−0.06	0.63	Sex, male vs. female	−0.07	0.55
Cohort	0.14	0.41	Cohort	0.15	0.38
per 1-SD increase of phosphate	−0.001	0.98	per 1-SD increase of phosphate	−0.006	0.91

per 1-SD increase of creatinine	0.07	0.32	per 1-SD increase of creatinine	0.12	0.06
per 1-SD increase of dialysis vintage	−0.01	0.86	per 1-SD increase of dialysis vintage	0.01	0.85

Abbreviations: dp-ucMGP, desphospho-uncarboxylated matrix Gla-protein; IndS, indoxyl sulfate; pCS, p-Cresyl sulfate; ESKD, end-stage kidney disease; 1-SD, one standard deviation; CCPB, calcium-containing phosphate binder.* $R^2 = 0.10$, # $R^2 = 0.09$.
